# An Adult Case of Gastric Volvulus Associated With Wandering Spleen: A Case Report

**DOI:** 10.7759/cureus.87114

**Published:** 2025-07-01

**Authors:** Hajar Slaoui, Ismail Chaouche, Zineb Ezzoulali, Hajar Ouazzani, Nizar El Bouardi, Badreddine Alami, Moulay Youssef Alaoui Lamrani, Meryem Boubbou, Mustapha Maaroufi

**Affiliations:** 1 Radiology Department, Hassan II University Hospital, Fez, MAR; 2 Mother and Child Radiology Department, Hassan II University Hospital, Fez, MAR

**Keywords:** abdominal surgical emergency, case report, ct scan, gastric volvulus, wandering spleen

## Abstract

Gastric volvulus is an uncommon condition characterized by the rotation of the stomach on its own mesentery. Wandering spleen (WS), defined by the excessive mobility of the spleen, is another rare entity. Although both conditions share a common cause, the absence or laxity of their supporting ligaments, their coexistence is rarely documented. Both can be life-threatening if left untreated. We report a case of an adult male patient presenting with intestinal obstruction secondary to gastric volvulus, in whom a WS was incidentally diagnosed.

## Introduction

Gastric volvulus is a rare type of volvulus that occurs when the stomach twists on its mesentery, which is an abdominal membrane that connects the intestines to the abdominal cavity, and encloses blood vessels, nerves, and lymphatic vessels supplying the intestines. It is a surgical emergency and can be fatal if left untreated due to strangulation [1]. Wandering spleen (WS) is also a rare condition, characterized by excessive splenic mobility and migration of the spleen to any abdominal or pelvic position [2]. The association of these two conditions is unusual, although they share a common etiology: the absence of an intraperitoneal visceral ligament, which is most commonly seen in children [2].

Though rare, gastric volvulus must always be considered when clinical symptoms are suggestive. Additionally, a WS, often asymptomatic, may be discovered in this context and may lead to splenic torsion. If not treated surgically in time, these entities can be potentially life-threatening. We are reporting this rare association in an adult presenting with intestinal obstruction, who underwent a laparotomy, with gastric detorsion, gastropexy, and splenopexy.

## Case presentation

A 58-year-old male presented to the emergency room complaining of acute colicky abdominal pain, vomiting, hiccups, three episodes of hematemesis, and signs of bowel obstruction. His medical history was unremarkable. The patient was conscious, had a blood pressure of 110/70 mmHg, a pulse of 85 beats per minute, and a body temperature of 37.5°C. Physical examination revealed abdominal distension, with splenomegaly extending to the left iliac region. Laboratory results are presented in Table 1.

**Table 1:** Laboratory investigations.

Parameters	On admission	Reference values
Total leukocytes (×10^3^/uL)	8.23	4.0-10.0
Hematocrit (%)	49.3	26-50
Hemoglobin (g/dL)	16.7	13-17
Platelet (×10^3^/uL)	137	150-410
Serum urea (g/L)	1.42	0.17-0.43
Serum creatinine (mg/L)	21	4-14
Serum potassium (mEq/L)	3.2	3.5-4.5
Serum sodium (mEq/L)	138	135-145
Serum total protein (g/L)	78	66-83
C-reactive protein (CRP) (mg/L)	31.2	0-5

**Figure 1:**
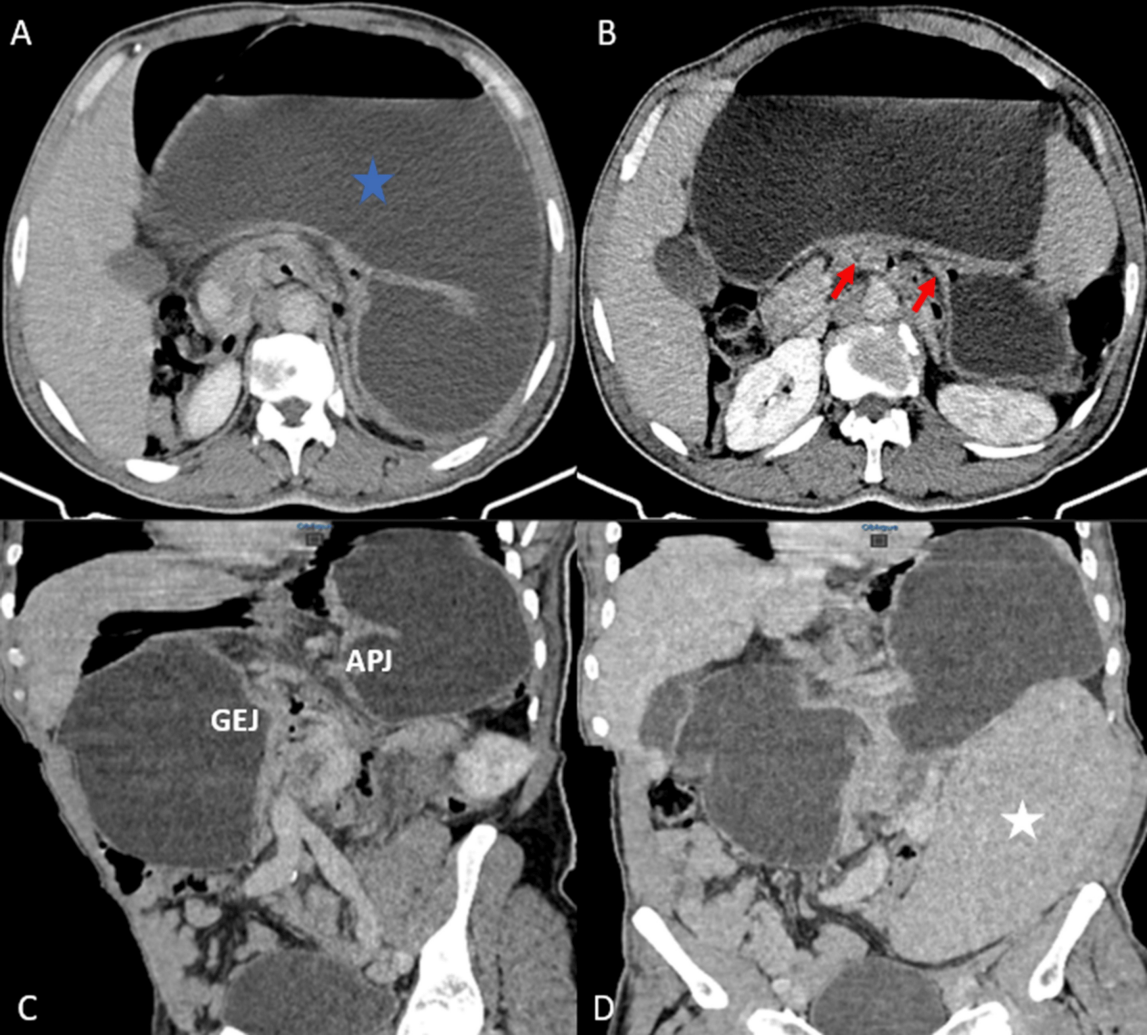
Contrast-enhanced abdominal CT findings. Grossly enlarged stomach (A, blue star). The stomach is twisted mesenteroaxially, showing the typical double bird beak sign (B, red arrows), an upside-down-looking stomach (C) with the APJ positioned higher than the GEJ. The enlarged spleen is positioned in the left lumbar and iliac region (D, white star). APJ: antropyloric junction, GEJ: gastroesophageal junction

An abdominal contrast-enhanced CT (Figure 1) revealed a grossly distended stomach and an upside-down stomach appearance with the antrum and pylorus above the fundus. There was no evidence of ischemia or perforation of the stomach. CT also showed an enlarged spleen (21 cm in length), positioned in the left lumbar and iliac region, normally enhancing. The diagnosis of an acute mesenteroaxial gastric volvulus was confirmed. The patient underwent an emergency laparotomy. The exploration revealed a markedly distended stomach, twisted but viable, without evidence of a hiatal hernia or diaphragmatic hernia. Gastric detorsion with gastropexy and splenopexy was performed.

**Figure 2:**
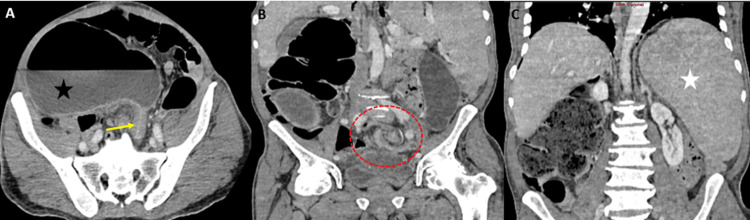
Postoperative contrast-enhanced abdominal CT findings: An important colonic distension (A, black star). A bird beak sign (A, yellow arrow) and a whirl sign with (B, red circle) indicating a mesentericoaxial sigmoid volvulus without any signs of digestive ischemia. A persistently enlarged spleen located in the left hypochondrium following splenopexy (C, white star).

Four days after the surgery, the follow-up was marked by clinical signs of bowel obstruction consisting of failure to resume bowel movement accompanied by abdominal distension. An abdominal CT scan (Figure 2) revealed an important colonic distension due to a mesentericoaxial sigmoid volvulus without any signs of digestive ischemia. Initially, an endoscopic detorsion was performed, followed by a laparotomy for a sigmoidectomy. Peroperative exploration revealed a mobile left colon with a dolichosigmoid and a mobile cecum. A sigmoidectomy with a colo-colic anastomosis and a cecopexy was performed. The postoperative course was uneventful. The patient remained asymptomatic at three-month follow-up.

## Discussion

Gastric volvulus refers to the rotation of the stomach on its mesentery by at least 180°. It has been classified into three types based on the rotation angle of the stomach axis [1,3]. The most common type is the organoaxial (60% of cases). The twisting occurs along its horizontal axis. The second is mesenteroaxial, occurring on the axis of the gastrohepatic mesentery, seen in around 26%, and the third, which is the least common, is a combination of the first two types [1,4]. Gastric volvulus is a surgical emergency that leads to bowel obstruction and impairment of vascularity and can be fatal if not treated.

Radiography, gastrointestinal (GI) fluoroscopy, and CT scan are the most frequently employed modalities to help diagnose gastric volvulus and its complications. Plain radiographs can show a distended stomach and the characteristic gastric double-bubble sign in the mesenteroaxial type, while in the organoaxial type, the lesser curvature appears to be under the greater curvature [2,5]. Upper GI fluoroscopy can show a distended stomach with or without its inversion, and a thin or absent passage of ingested contrast material into the duodenum. CT scan has the highest sensitivity and specificity in confirming acute gastric volvulus, with a reported 90% overall accuracy [6]. The stomach appears horizontal with the greater curvature superior to the lesser curvature, and a downward pointing of the pylorus in the organoaxial type. A vertical stomach with pylorus superior to the fundus is seen in the mesenteroaxial type. A CT scan can also reveal an associated hiatal hernia and help identify complications, such as ischemia and perforation, by showing gastric wall edema, poor gastric wall enhancement, perigastric fluid, pneumatosis, pneumoperitoneum, and pleural effusion [6]. Ultrasound is not usually used to diagnose gastric volvulus, but it can be the first imaging modality performed in the emergency department. It can show a distended, fluid-filled stomach and peritoneal fluid as a sign of bowel wall compromise.

WS accounts for only 0.2% of cases, mostly in children and adult women of childbearing age [7]. It can be congenital or acquired. Congenital WS is due to the absence or maldevelopment of the spleen’s supporting ligaments: gastrosplenic and splenorenal ligaments. Acquired WS can be caused by predisposing factors such as hormonal effects of pregnancy and abdominal wall laxity that can weaken these ligaments [7,8]. The presentation of WS is variable. It can remain asymptomatic throughout life or present in several clinical forms of varying severity. The elongated splenic vascular pedicle can twist, causing congestion and splenomegaly, which was the case in our patient. Depending on the severity of the torsion, complications such as splenic infarction, gangrene, and spleen rupture can occur. Other complications can be seen due to the hypermobility and the mass effect of a WS, like gastric volvulus, intestinal obstruction, and pancreatitis, with rare associations to portal hypertension and gastric varices [9]. Although WS and gastric volvulus share a common cause, their coexistence is not frequently described in the literature [4].

The management of gastric volvulus relies mainly on surgical treatment by performing stomach detorsion and gastropexy, either via laparotomy or laparoscopy. Sometimes, a subtotal or total gastrectomy is needed in the presence of gangrene [1,4]. In cases of association with a WS, performing a splenopexy is essential to avoid future complications [1]. Following radiological and surgical investigations, we suspected that our patient had peritoneal attachment abnormalities, which were responsible for visceral hypermobility (WS and a mobile caecum) and digestive torsions (gastric and sigmoid volvulus).

Acute gastric volvulus can be associated with a high morbidity rate, especially organo-axial volvulus or in cases of underlying cardiorespiratory comorbidities. In series reporting chronic gastric volvulus, the morbidity rate is negligible or even zero [10,11]. The recurrence rate of gastric volvulus varies considerably from one series to another, ranging from 0% to 30%. Different therapeutic approaches can explain this variability, as well as the patient population in which the volvulus occurs, and the wide disparity in follow-up duration across these series. However, to date, no prospective randomized comparative studies confirm and validate these risk factors [10].

## Conclusions

Although WS and gastric volvulus share a common cause, their coexistence is not frequently described in the literature. Patients diagnosed with acute or chronic gastric volvulus should be screened for WS with an abdominal CT scan, in order to undergo a prophylactic splenopexy to avoid complications and relapses.
